# Supplementary material for: Social behavioural adaptation in Autism
Source: PLoS Comput Biol. 2020 Mar 16;16(3):e1007700. doi: 10.1371/journal.pcbi.1007700 (PMC7108744; doi:10.1371/journal.pcbi.1007700)
Supplement: S1 Text — (DOCX) [file pcbi.1007700.s001.docx]

This document provides supplementary information regarding: the experimental protocol (section 1), the credibility of the framing manipulation (section 2), the effect of motivational manipulations on the game's performance (section 3), analysis of behindhand response rates (section 4), model-free Volterra decompositions of trial-by-trial choice sequences (section 5), differences in adaptation strategies between AS and NT participants (section 6), the impact of the framing manipulation on the repertoire's flexibility (section 7), the statistical relationships between computational phenotypes (section 8), and their relationship with performance in the game (section 9).

1. **The experimental protocol**

The experimental protocol was adapted from a previous computational study on healthy adults [3]. In brief, participants are either led to believe that they are competing online against somebody else (*social* framing condition), or that they are playing a gambling game (*non social* framing condition).

At the beginning of each task, instructions were displayed on the screen and read out loud by the experimenter. In order not to bias results with additional social motivational effects [4], the experimenter left the room during the test phase of each task. Responses were given on a special pad with three buttons: left, right and enter. Participants were paid 15$ per hour, with an additional performance bonus corresponding to a randomly drawn session (see section 3 of this document). The order of all task factors was counterbalanced across participants.

To support our deceptive manipulation in the social framing condition, we used the following cover story (which was approved by the ethical committee). Participants were told that the study was performed in collaboration with other research centres and that they would play on an online internet platform with other people. When the "hide-and-seek" game started, a mock-internet connection screen appeared for a few seconds with dummy IT information appearing gradually in English, such as the number of connected sites. Then, a screen appeared, indicating (in French) that the connection was successful, and that there were 17 other players online. Participants could then use a pseudonym (NB: we told them not to use their full name for anonymity reasons). The game's instructions were then displayed on the screen, at the end of which participants were told that they were randomly assigned the role of "seeker" in the game. Before each game session, the mock online system would first ask participants if they were ready to play, and then waited "until an online hider was ready" while indicating "seeker [participant's pseudo] is ready". Finally, the hiders' pseudonym appeared and the game began.

In the non-social framing condition, participants were simply told to think of the game as a gamble, in which earnings were depending on their ability to correctly guess the trial-by-trial outcomes. No other specific instruction was provided.

Note that the deceptive manipulation was privately disclosed at the end of the experiment.

1. **Was the framing manipulation similarly credible in both AS and NT groups?**

We assessed the credibility of our social versus non-social framing manipulation using dedicated debriefing questionnaires. In particular, we evaluated whether AS and NT participants were similarly deceived in believing that they were playing against other human subjects in the social condition. We first included questions designed to indirectly assess whether participants attributed an intentional strategy to their opponent: Q1: "did you feel that your opponents were playing randomly?", Q2: "did you feel that your opponents were taking your actions into account?", Q3: "did you feel that your opponents were trying to predict your actions?". For each question, participants were asked to report subjective ratings using Lickert scales (range: 0-10) and provide an open-ended verbatim report. For the latter, we evaluated the frequency of explicit references to (i) randomness in the opponent’s behaviour, (ii) regularity/pattern detection, (iii) mental states’ attributions, and (ii) doubts and/or suspicions regarding the deceptive manipulation. We found no significant difference between AS and NT groups, neither in terms of quantitative rating scales (Q1: AS: 5.6±1.7, NT: 6.1±2.0, F(1,45)=0.22, p=0.36, R^2^=0.5% ; Q2: AS: 5.5±2.1, NT: 6.6±1.6, F(1,45)=0.73, p=0.60, R^2^=1.6% ; Q3: AS: 5.9±1.5, NT: 6.4±1.3, F(1,45)=0.93, p=0.66, R^2^=2.0%), nor in terms of verbatim analysis (results not shown). Typical verbatim answers would be: "I am sure they were like me" (NT), "I could feel an intention behind the choices" (NT), "people anticipated that I would switch back and forth" (NT), "I suppose they did not want not be found, therefore they were going against what they thought I would do" (NT), "surely, they were thinking" (AS), "they were trying to see whether I was following some strategy" (AS), "of course they were trying to predict my actions, since this was the goal of the experiment" (AS). We note that only two participants (1 NT and 1 AS) explicitly suspected that they were playing against some sort of algorithm or machine.

Finally, we asked participants their opinion about the objective of the experiment (Q4). Typical verbatim answers to this question would be: "knowing if we are smart enough to guess others' actions" (NT), "outsmart our opponents" (NT), "evaluate our patience or focus?" (NT), "evaluate something like empathy" (NT), "testing the understanding of others" (AS), "testing how fast I am on gambling tasks" (AS), "predicting the other person" (AS), "to let go, given that one cannot find any logic in randomness" (AS). We note that peoples' intuitions about the goal of the experiment were very variable, without any strong qualitative difference between the two groups.

1. **Do financial incentives impact on task performance?**

For the sake of simplicity, we described our experiment in terms of a 4x2x2 factorial design, with 4 opponent types (RB, *0-ToM*, *1-ToM* and *2-ToM*), 2 framing conditions (social versus non-social) and 2 repetitions of each game session. These repetitions however, were associated with 2 reward conditions that varied in terms of the financial payoff attached to a correct answer in the games. More precisely, in the high (resp., low) reward condition, the maximal payoff that participants could earn over one game session was 10$ (resp., 1 cent). We introduced this incentive manipulation in the aim of assessing whether variations in task performance could be due to motivational effects. This is particularly relevant for the framing effect, which could in principle result from differences in intrinsic reward when playing against other humans or not. In other words, people may perform better in the social condition because they have more fun, and thus allocate more mental effort to the game. Such potential motivational effects are important, given that the main difference between AS and NT groups is that only the latter show a significant framing effect. In line with motivational theories of autism [5], one could thus argue that AS participants show relative performance deficits when playing against other humans because they are not as interested as NT controls. However, when analyzing task performance, we found no significant effect of incentive nor any interaction with the two other task factors (all p>0.3). Moreover, when focusing on the AS group only, there was no main effect of incentive (p=0.24) nor any interaction with opponent types (p=0.19).

At this point, one may argue that subjects, and in particular AS participants, may not be sensitive to such incentive manipulation. This is the reason why we asked all participants to perform a motivation control task, whose aim was to assess the impact of the same incentive manipulation on performance in a simple cognitive control task. In brief, we asked participants to perform a sequential numerical Stroop task with two different monetary incentives (high reward: 10$, low reward: 1 cent). This task was adapted from a previous study on the motivation of mental and physical effort [6]. The potential payoff was displayed on the screen for 3500 msec. Then a virtual ladder appeared on the screen, with a pair of digits shown next to each graduation (see Figure A1 below). The two digits differed in their numerical magnitude (from 1 to 9) and in their physical size (a big font and a smaller font). Participants were instructed to press the button corresponding to the side with the biggest font size. Correct responses were made either congruent or incongruent with numerical comparisons. They had to respond as quickly as possible and progressed from a comparison to the next in a sequential manner. To prevent participants from developing strategies such as responding randomly as fast as possible, we introduced a refractory period after an incorrect button press during which no response was taken into account (this lasted for 25% of the maximal time allocated for a trial). After a successful response, the cursor on the ladder went up to the following graduation. The effective earning (i.e. the proportion of correct answer times the maximal payoff) was displayed at the end of the trial (after the time limit or after reaching the top of the ladder).


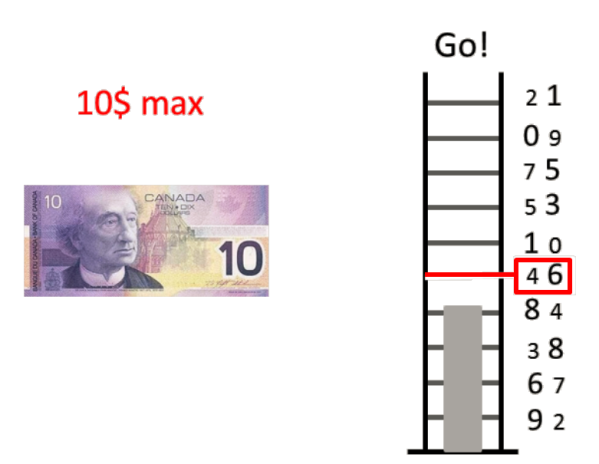


**Figure A1: Example trial of the motivational Stroop task.** The reward condition (here: high incentive) is indicated on the left of the screen. The ladder on the right side of the screen indicates how many successful responses were given so far. In addition, the current (here congruent) comparison is highlighted in red.

Performance in the Stroop task was measured in terms of the average number of correct answers. We measured the impact of the incentive manipulation in terms of the performance difference between the high and low reward condition, for each subject. The effect was significant either when pooling the two groups together (t[47] = 4.1, p=0.001, R^2^=26.3%) or when considering the two groups independently of each other (NT: t[23]=2.6, p=0.01, R^2^=22.7% ; AS: t[23]=3.7, p=0.01, R^2^=37.3%). Note that a 2-sample t-test revealed no difference between the NT and AS groups (F[1,45] =0.25, p=0.38, R^2^=0.5%).

In conclusion, although both NT and AS participants modulate (to the same extent) their mental effort as a function of external financial rewards (as shown by the control Stroop task results), such incentive manipulation had no impact whatsoever on performance in the main task (i.e. "hide-and-seek" and "gambling" games). Motivational factors (e.g., task engagement or focus) are thus unlikely confounds for observed performance differences between NT and AS groups. Note that this null result is also the reason why, in the main manuscript, we simplify the description of our experimental design and consider the two incentive conditions as simple repetitions of framing and opponent combinations.

1. **Can reaction times confound performance patterns?**

In the main text, we show that AS individuals perform worse than controls against mentalizing agents in the social framing condition. One may ask whether this may not in fact be due to the fact that AS individuals are typically slower than neurotypic people. This would be because in the task, participants have only 1.3 second to respond, which would potentially be too short for AS individuals to reach the correct decision. The ensuing "behindhand errors" could then confound analyses of performance data. In particular, this would explain performance differences in situations where strategic thinking is (in principle) most needed, i.e. against mentalizing agents in the social framing condition.

We thus looked at behindhand response rates (i.e. trials whose RT reaches the decision time limit), and how these vary according to group, framing and opponents. This is summarized in Figure A2 below.

|  |  |
| --- | --- |

**Figure A2: Behindhand response rates.** The percentage of responses whose timing has reached the 1.3 sec limit (y-axis) is plotted as a function of opponent types (x-axis) and framing conditions (blue: social framing, red: non-social framing). *Left*: AS group. *Right*: NT group.

Note that the average rate of behindhand responses is very low (2.7% for the NT group and 3.0% in the AS group). Now, one can see that both groups show very similar behindhand response patterns. In particular, in both groups, the rate of behindhand responses in the social framing condition seems to be lower than in the non-social framing condition (AS group: p=0.06, NT group: p=0.008). There is, however, no effect of opponent (AS group: p=0.98, NT group: p=0.62), nor any framing×opponent interaction (AS group: p=0.92, NT group: p=0.38). In addition, post-hoc tests show that the behindhand response rate of AS individuals is not higher against mentalizing agents than against non-mentalizing agents in the social condition (p=0.75). Finally, there is no significant main effect of the group, nor any significant interaction with any task factor. Taken together, the pattern of behindhand responses is thus globally inconsistent with the performance pattern reported in the main text (cf. Figure 2).

1. **Volterra decompositions of trial-by-trial choice sequences**

Interpreting performance patterns (across conditions) in terms of covert cognitive strategies is difficult, essentially because different strategies in the game may eventually yield similar performances. This motivates Volterra decompositions [7] of participants' choice sequences, which look at how much trial-by-trial variance in choice sequences can be concurrently explained by the past history of both players actions. In particular, we use Volterra decompositions to capture peoples' strategies in terms of model-free mixtures of imitative and perseverative tendencies [3,8]. More precisely, we performed the following session-specific Bayesian logistic regression:

$\begin{matrix} p\left(a^{self}\left|\omega\right.\right)=\prod_{t} q_{t}\left(\omega\right)^{a_{t}^{self}}\left(1-q_{t}\left(\omega\right)\right)^{1-a_{t}^{self}} \\ q_{t}\left(\omega\right)=s\left(\omega^{0}+\sum_{\tau} \omega_{\tau}^{op} \left(2a_{t-\tau}^{op}-1\right)+\sum_{\tau} \omega_{\tau}^{self} \left(2a_{t-\tau}^{self}-1\right)\right) \end{matrix}$ (A1)

where $q_{t}\left(\omega\right)=p\left(a_{t}^{self}=1\left|\omega\right.\right)$ is the probability that the agent chooses the first option $a_{t}^{self}=1$ at trial $t$ , $\tau$ is some arbitrary time lag and $\omega$ is the so-called Volterra kernel ($\omega^{0}$ is a potential bias for the first option $a^{self}=1$). Volterra kernels $\omega^{op}$ (resp. $\omega^{self}$) capture the impact of lagged opponent's (resp. own) actions $a^{op}$ (resp. $a^{self}$) onto peoples' choice probability. In other terms, $\omega^{op}$ and $\omega^{self}$ capture imitative and perseverative tendencies, respectively. For the sake of statistical efficiency, we constrain the Volterra kernels to parameterized exponential mappings, i.e.: $\omega_{\tau}=A\exp\left(-\lambda\tau\right)$, where $A$ and $\lambda$ are the kernel's magnitude and temporal decay, respectively. For each individual and each game session, we fit the resulting model and report the kernels' magnitudes $A^{op}$ and $A^{self}$at the group level [9]. Figure A3 below summarizes the Volterra decomposition in terms of both mean Volterra kernels' magnitudes, for all conditions and both groups.


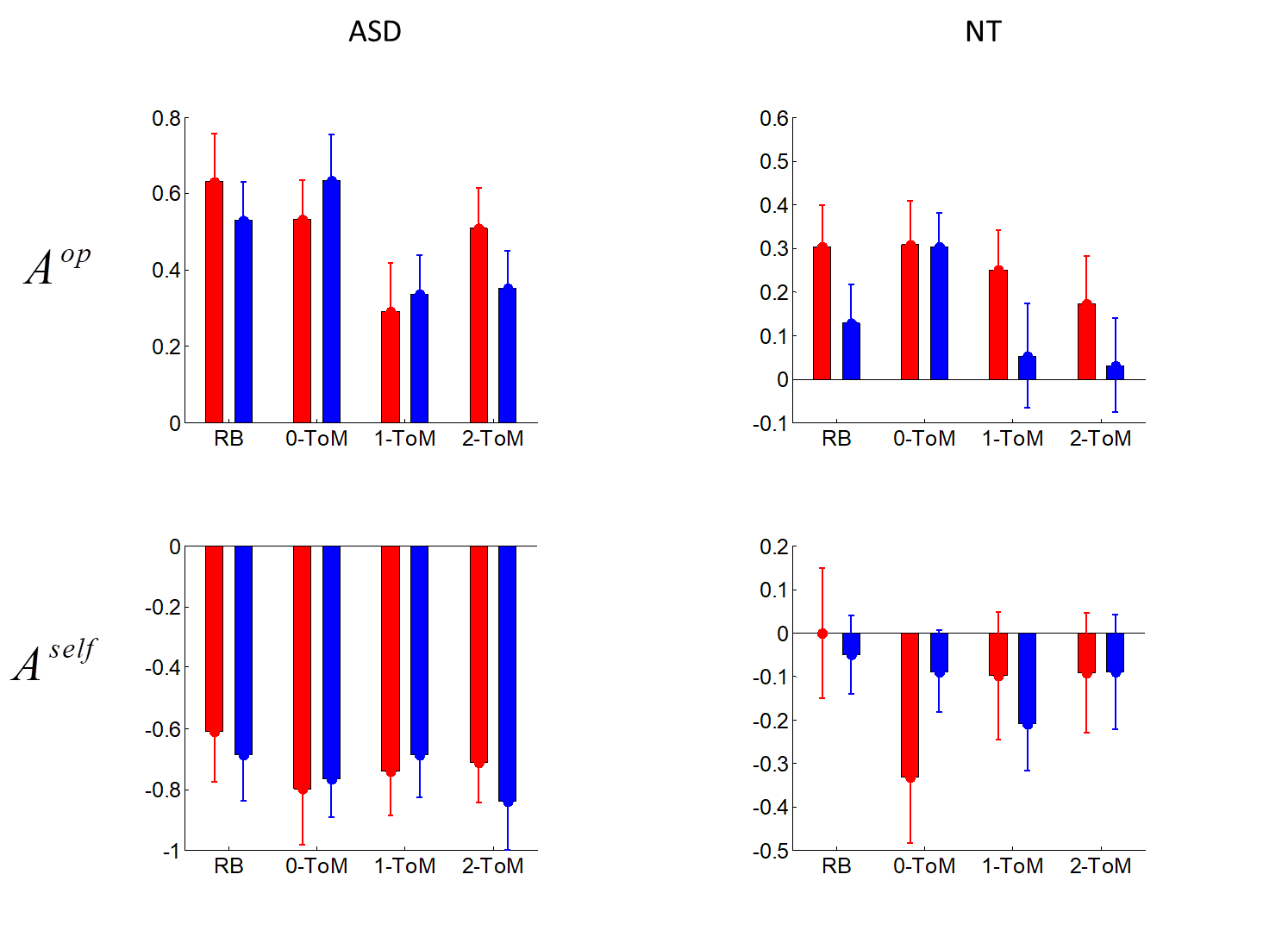
 **Figure A3: Volterra analyses.** Volterra kernels’ magnitudes (y-axis) are plotted as a function of opponent types (x-axis) and framing conditions (blue: social framing, red: non-social framing). Note that within-subject estimates of Volterra kernel magnitudes have been averaged over repetitions. *Top*: imitative kernel magnitude $A^{op}$. *Bottom:* perseverative kernel magnitude$A^{self}$. *Left*: AS group. *Right*: NT group.

We performed pooled-variance ANOVAS for *A_o_* (magnitude of the Volterra kernel of opponent's actions, which signals imitation tendencies) and *A_s_* (magnitude of the Volterra kernel of the agent's own actions, which signals perseveration tendencies) separately. For *A_s_*, we found a very strong main effect of group (F[3,690]=85, p<10^-4^, R^2^=10.9%) but no other main effect or interaction. For *A_o_*, we also found a very strong effect of group (F=226, p<10^-4^, R^2^=24.7%), an effect of opponent (F=7.9, p<10^-4^, R^2^=1.1%) and an effect of framing (F=4.0, p=0.046, R^2^=0.6%). We then looked at both groups separately. In the control group, only the framing effect was significant (p=0.03); there was no main effect of opponent (p=0.11) and no interaction (p=0.14). In the AS group, there was a strong effect of opponent (p<10^-4^), but no effect of framing (p=0.49) nor any interaction (p=0.56). This is a first indication that, in contrast to control participants, AS participants did not change their strategy according to whether they were playing against a human opponent or not (although they adapt to different opponents).

At this point, we asked whether we could predict the diagnosis label (AS versus NT) based upon Volterra kernel magnitudes across opponent types and framing conditions (averaged over repetitions). A leave-one-out classification scheme based upon logistic regression eventually yields a cross-validated classification accuracy of 67%, which is significant (p=0.02). In other words, AS and NT participants can be discriminated based upon their adaptation strategies, as captured by agnostic Volterra decompositions of trial-by-trial action sequences.

Finally, we asked whether the effect of group, framing and opponent onto performance were mediated by changes in strategy (as captured by Volterra kernels). We thus computed the correlation between the estimated Volterra kernel of each individual's choice sequence in each condition and that of the corresponding optimal adaptation strategy (namely: 0-ToM against *RB*, [k+1]-ToM against *k-ToM*). Classical Sobel mediation tests [10] then confirmed that the three-way interaction [groups × opponents × framing] was mediated by differences in similarity to best response (p<10^-4^). These results are important, because they indicate that performance variations are driven by differences in group-specific strategies. One cannot, however, directly interpret quantitative changes in Volterra kernels in terms of qualitative differences in adaptation strategies. Evidence for the latter can only be derived from direct quantitative comparisons of peoples' trial-by-trial choices sequences and predictions derived from learning models. The first steps of this type of model-based analysis are summarized in the next section of this document. Finalized computational phenotypes are reported in the main text.

1. **Do AS and NT participants use different adaptation strategies?**

Our statistical data analysis proceeds in three steps of increasing specificity, namely: multiple regressions of behavioural performances (cf. ANOVA in the main text), Volterra decompositions of trial-by-trial choice sequences (cf. previous section of this document) and Bayesian model comparison of adaptation strategies. In this section, we provide further details regarding the latter approach. For each subject, we inverted the eight learning/decision models described in the main text using a variational bayesian approach [11,12]. Eventually, we obtained 8x48x2x4x2=6144 model evidences (8 models, 48 participants, 2 framing conditions, 4 opponent conditions and 2 repetitions). We then performed a group-level random-effect Bayesian model comparison (i.e. RFX-BMC), which accounts for the possibility that different subjects may be best described by distinct models [13,14].

To begin with, one may ask whether learning models are identifiable from each other, i.e. how often Bayesian model comparison confuses learning models with each other. This can be assessed by simulating trial-by-trial action sequences under each learning model, comparing models given each simulated data, and then assessing model selection errors in terms of a confusion matrix. The results of such confusion analysis has already been reported elsewhere [3]. In brief, under our experimental design, the overall identifiability of the learning models included in the comparison set is very satisfactory. In particular, we do not expect any systematic comparison bias nor lack of discriminability.

First, we asked whether there are group differences in terms of the repertoire of adaptation strategies that people exhibit in both the social and non-social framing conditions. For the sake of simplicity, we summed log model evidences over opponents and repetitions, to derive (fixed-effect) estimates of model evidences for each participant, in each framing condition. Figure A4 below shows the results of RFX-BMC analyses performed separately for social and non-social framing conditions.


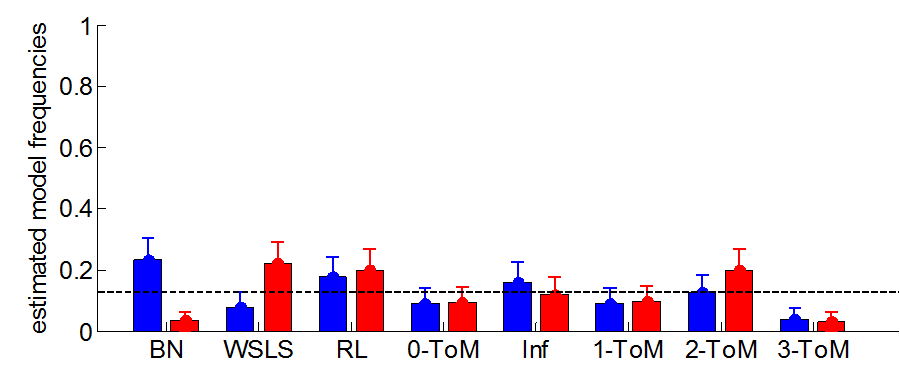


**Figure A4: RFX-BMC for the control (NT) group.** Estimated model frequencies (y-axis ; ±bayesian s.e.m.) are plotted for each model (x-axis) for both the social (blue colour) and non-social (red colour) framing conditions. The dotted black line indicates chance level (i.e. identical frequency for all models).

One can see that no learning model clearly stands out for the NT group. In fact, Bayesian omnibus risks show that the model frequency profiles of Figure A4 do not deviate strongly from expectations derived under chance (social framing: BOR=0.96, non-social framing: BOR=0.44). This does not imply that models do not fit trial-by-trial NT participants’ data. Recall that we had included a chance model in the comparison set, namely: “Biased Nash” or BN. This model reduces trial-by-trial choice data to a sequence of (possibly biased) binomial random samples. This is as close a null model as it gets. However, it very rarely wins the within-subject model comparison, since its average frequency is below 20%. In other terms, other models are better than chance in about 80% of the cases. Rather, high BORs imply that RFX-BMC cannot conclude on any winning learning model. Note that this null finding is not inconsistent with the fact that NT participants' learning repertoire exhibit high flexibility across conditions.

This contrasts with the estimated model frequencies for the AS group, which are depicted on Figure A5 below.


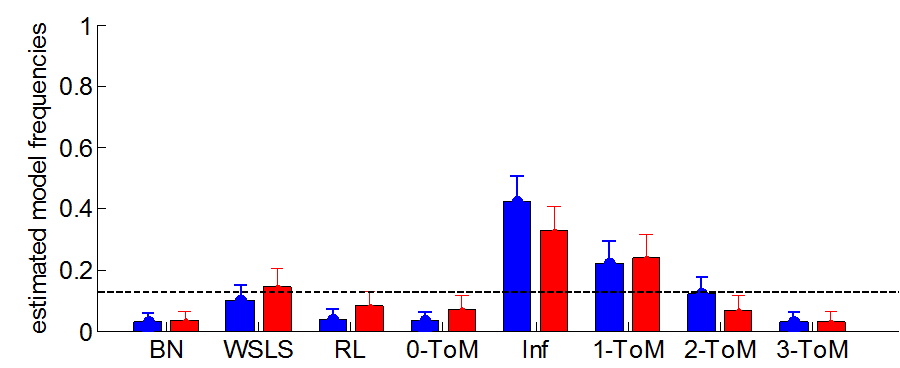


**Figure A5: RFX-BMC for the AS group.** Same format as Fig. A4.

In this case, Bayesian omnibus risks show that model frequency profiles in the AS group are unlikely to be driven by chance (social framing: BOR=0.0001, non-social framing: BOR=0.01). In addition, a between-condition RFX-BMS analysis [13] concludes that a difference in model frequencies between framings is unlikely (Protected Exceedance Probability: PEP=0.18). Taken together, this means that one can think of the covert strategy of the AS group as being best described in terms of the '*influence learning'* model, irrespective of the framing condition.

Finally, we asked whether we could predict the diagnosis label (AS versus NT) based upon within-subject learning models’ attributions. Recall that we have derived the log-evidence of each learning model, for each subject, in each experimental condition. First, we marginalized over experimental factors by summing the log model evidences over all conditions. This yielded a feature space of 8 dimensions (8 models), which was then fed to a leave-one-out classification scheme based upon logistic regression. It achieved a significant cross-validated classification accuracy of 69% (p=0.006). Second, we conditioned upon framing, by summing the log model evidences only over opponent and repetitions. This induced a feature space of 8×2 dimensions (8 models, 2 framing conditions), eventually yielding an improved classification accuracy of 71% (p=0.002). Note that pooling model evidences with performance patterns did not improve cross-validated classification accuracy. In brief, one can guess the diagnosis label from the model that best describes a given participant’s trial-by-trial choice data.

1. **Does the framing impact on the repertoire's flexibility between repetitions?**

In the main text, we report results regarding the sophistication and flexibility of peoples' behavioural repertoire. In particular, we show that the repertoire's flexibility (both between repetitions and between framings) is significantly reduced in the AS group. Here, we ask whether the framing itself impacts on the repertoire's flexibility between repetitions. Here, we measure, for each participant and each framing condition, the flexibility between repetitions (i.e. the posterior probability $\hat{f}$ that participants employ different adaptation strategies). The group-average results are shown in Figure A6 below:

**
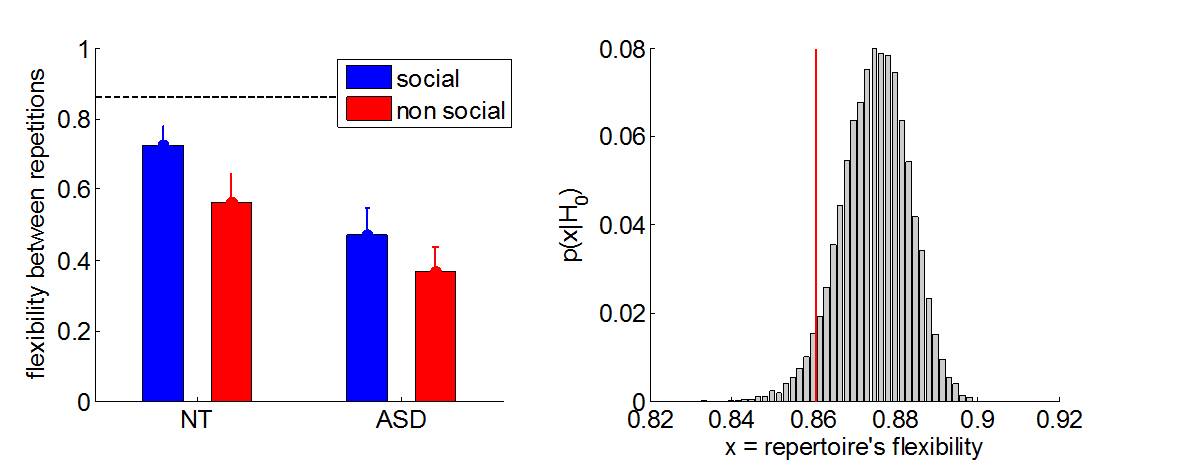
Figure A6: Flexibility between repetitions.** *Left*: Flexibility between repetitions (y-axis) is plotted as a function of group (NT versus AS) and framing conditions (blue: social framing, red: non-social framing). The black line indicates the 5% quantile of the flexibility score under the null. *Right:* the distribution of the flexibility score under the null (i.e. when model attributions are entirely random). The red line indicates the 5% quantile, i.e. the threshold under which flexibility is significantly below expected chance level.

A simple ANOVA shows no evidence for an interaction between framing and group (F[1,92]=0.19, p=0.67, R^2^=0.2%), a trend for a framing effect (F[1,92]=3.52, p=0.06, R^2^=3.7%) and a significant effect of group (F[1,92]=10.1, p=0.002, R^2^=9.8%). This justifies the simple analysis reported in the main text, which focuses on the group effect of flexibility between repetitions (marginalized over framing conditions). Now posthoc tests show that the framing effect is mostly driven by the NT group, i.e. framing has a significant effect in the NT group (F[1,23]=5.35, p=0.03, R^2^=18.9%) but not in the AS group (F[1,23]=1.37, p=0.25, R^2^=5.6%).

1. **Are computational phenotypes correlated across subjects?**

In the main text, we show that AS and NT participants differ in terms of their repertoire's sophistication and flexibility (both between framings and repetitions). This is interesting, provided sophistication and flexibility of people's learning repertoire really capture distinct features of peoples' cognitive style. We thus asked whether these measures were correlated across participants, above and beyond the group effect. Figure A7 below shows the correlation matrix between all computational scores, for each group separately.

**
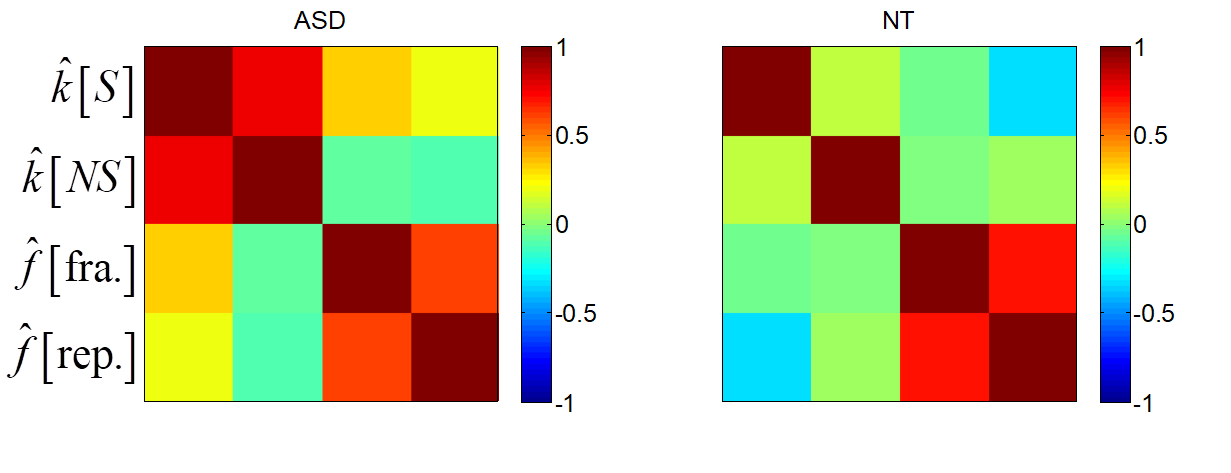
**

**Figure A7:** **Inter-individual co-variations of computational phenotypes**. Pairwise correlations (across participants) between scores of ToM sophistication (in both social and non-social framings) and repertoire's flexibility (across framings and across repetitions) for the AS (left) and NT (right) group.

After correcting for multiple comparisons, there is a significant correlation between measures of flexibility in both groups (AS: r=0.60, p=0.002, NT: r=0.71, p<10^-4^), and a significant correlation between measures of ToM-sophistication only in the AS group (r=0.75, p<10^-4^). None of the other pairwise correlations is significant (AS: p>0.13, NT: p>0.11). The likely implication of this analysis is twofold: (i) the repertoire's sophistication and flexibility are probably driven by independent mechanisms, and (ii) the repertoire’s flexibility may be thought of as an idiosyncratic trait (invariant of the measurement method).

Note that the relatively high correlation between both measures of AS participants’ ToM-sophistication is not surprising, given their subnormal repertoire’s flexibility (cf. Figure 4 in the main text).

1. **Are computational phenotypes related to performance in the game?**

Recall that our computational phenotypes are features of peoples’ behavioural repertoire, which is composed of a set of learning models (namely: *BN*, *WSLS*, *RL*, *0-ToM*, *Inf*, *1-ToM*, *2-ToM*, *3-ToM*). Numerical simulations actually show that each adaptation strategies produces a specific performance pattern across opponent types in the game. This is shown on Figure A8 below:


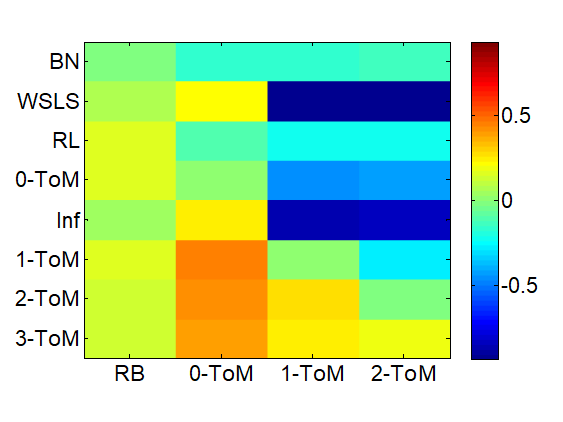


**Figure A8:** **Performance patterns of adaptation strategies**. The colormap encodes the net rate of correct answers (Monte-Carlo average over 5000 simulations) of each learning model (y-axis) against each opponent type (x-axis). NB: the *influence learning* model, which is the most likely adaptation strategy of AS participants (irrespective of the framing cf. Figure A4) wins over RB and 0-ToM, but looses against 1-ToM and 2-ToM. This is consistent with the raw performance results of AS participants (cf. Figure 2 in the main text).

One can see that the expected performance of *k-ToM* models increases with their ToM-sophistication. This implies that we expect a positive relationship between the repertoire’s ToM-sophistication $\hat{k}$ and observed performance in the game. Importantly, one would not expect any systematic relationship between the repertoire's flexibility and raw performance in the game. This is because the tendency to change one's strategy is as likely to bring performance improvements (e.g. from *WSLS* to *2-ToM*) as performance decrements (e.g. from *2-ToM* to *WSLS*). Having said this, the repertoire's flexibility will determine the magnitude of performance differences induced by experimental conditions (e.g., the framing effect).

1. **Are computational phenotypes related to other biographical and clinical variables?**

In addition to the main task, all participants were asked to perform an additional WAIS IQ test, and to fill-in clinical questionnaires, namely: BDI (depression symptoms' severity) and anhedonia self-rating scales (social and physical). No pairwise correlations between clinical variables and computational phenotypes survived correction for multiple comparisons. Of anecdotal interest is the observation that people's ToM-sophistication in the social framing of the game $\hat{k}\left[S\right]$ significantly increases with age (r=0.31, p=0.03).

**References**

1. Turing AM. Computing Machinery and Intelligence. Parsing the Turing Test. Springer, Dordrecht; 2009. pp. 23–65. Available: https://link.springer.com/chapter/10.1007/978-1-4020-6710-5_3

2. Saygin AP, Cicekli I, Akman V. Turing Test: 50 Years Later. Minds Mach. 2000;10: 463–518. doi:10.1023/A:1011288000451

3. Devaine M, Hollard G, Daunizeau J. The social Bayesian brain: does mentalizing make a difference when we learn? PLoS Comput Biol. 2014;10: e1003992. doi:10.1371/journal.pcbi.1003992

4. Chevallier C, Parish-Morris J, Tonge N, Le L, Miller J, Schultz RT. Susceptibility to the audience effect explains performance gap between children with and without autism in a theory of mind task. J Exp Psychol Gen. 2014;143: 972–979. doi:10.1037/a0035483

5. Chevallier C, Kohls G, Troiani V, Brodkin ES, Schultz RT. The Social Motivation Theory of Autism. Trends Cogn Sci. 2012;16: 231–239. doi:10.1016/j.tics.2012.02.007

6. Schmidt L, Lebreton M, Cléry-Melin M-L, Daunizeau J, Pessiglione M. Neural Mechanisms Underlying Motivation of Mental Versus Physical Effort. O’Doherty JP, editor. PLoS Biol. 2012;10: e1001266. doi:10.1371/journal.pbio.1001266

7. Brockett RW. Volterra series and geometric control theory. Automatica. 1976;12: 167–176. doi:10.1016/0005-1098(76)90080-7

8. Devaine M, San-Galli A, Trapanese C, Bardino G, Hano C, Jalme MS, et al. Reading wild minds: A computational assay of Theory of Mind sophistication across seven primate species. PLOS Comput Biol. 2017;13: e1005833. doi:10.1371/journal.pcbi.1005833

9. Daunizeau J, Adam V, Rigoux L. VBA: A Probabilistic Treatment of Nonlinear Models for Neurobiological and Behavioural Data. PLoS Comput Biol. 2014;10: e1003441. doi:10.1371/journal.pcbi.1003441

10. Sobel ME (Ed ). Asymptotic Confidence Intervals for Indirect Effects in Structural Equation Models. Sociol Methodol. 1982;13: 290–312.

11. Daunizeau J. On parameters transformations for emulating sparse priors using variational-Laplace inference. ArXiv170307168 Q-Bio Stat. 2017; Available: http://arxiv.org/abs/1703.07168

12. Friston K, Mattout J, Trujillo-Barreto N, Ashburner J, Penny W. Variational free energy and the Laplace approximation. NeuroImage. 2007;34: 220–234. doi:10.1016/j.neuroimage.2006.08.035

13. Rigoux L, Stephan KE, Friston KJ, Daunizeau J. Bayesian model selection for group studies - revisited. NeuroImage. 2014;84: 971–985. doi:10.1016/j.neuroimage.2013.08.065

14. Stephan KE, Penny WD, Daunizeau J, Moran RJ, Friston KJ. Bayesian model selection for group studies. NeuroImage. 2009;46: 1004–1017. doi:10.1016/j.neuroimage.2009.03.025
