# Supplementary material for: Social behavioural adaptation in Autism
Source: PLoS Comput Biol. 2020 Mar 16;16(3):e1007700. doi: 10.1371/journal.pcbi.1007700 (PMC7108744; doi:10.1371/journal.pcbi.1007700)
Supplement: Attachment — Submitted filename: response to reviewers.docx [file pcbi.1007700.s002.docx]

To begin with, we would like to thank the reviewers for their constructive criticism, which, we think, has enabled us to significantly improve our manuscript. We have tried to address each of your comments: you will find below a point by point response (in addition to the changes we made to the previous version of the manuscript, when relevant).

**Editor's comments:**

*If I were to pick a theme which is most important to address, is the one of validity and subsequent diagnostic value of the method advocated here. As far as using this test in a clinical setting is concerned, the analysis that this manuscript present does not improve on the accuracy (hence scientific validity) of clinician diagnosis, and in fact this is impossible if the latter is taken as the measure by which the task-based diagnosis is judged. Of course this is most problematic if such tasks are seen as stand-alone assessments; They may indeed be very useful as screening tools (with the caveat that they then have to be tested against much more straightforward self- and collateral- reported questionnaires).*

We thank you for your kind and patient evaluation. We agree with you that expecting a test to improve on the accuracy of a diagnostic is doomed to fail, if the reference point is the diagnosis itself. That our test can achieve 79% of accuracy in diagnostic prediction is only relevant for the purpose of comparing this test with other tests of the same kind, or as a proof-of-concept demonstration. In fact, we would argue that the long-term goal of approaches of this kind is not to reflect the diagnosis per se, but rather to guide clinical decisions. Ideally, a useful approach should reflect a pathological mechanism and predict outcome and treatment response. Diagnosis is just a proxy for prediction, and we have to admit that current categorical diagnoses are not quite satisfactory in this regard (Insel 2013^[1]^). In turn, evaluating the clinical utility of our approach would require assessing how it relates to genetic variants, brain metrics, specific outcomes or response to intervention. This is beyond the scope of the current work, but we intend to pursue these issues in forthcoming publications (on a different population sample). As you will see, we have modified our manuscript to tone down our clinical claims. We hope that you will find these modifications render our work suitable for publication in PLoS CB.

**Response to Reviewer #1:**

*In this excellent ms. the authors ambitiously suggest to "decompose reciprocial social interactions into their computational constituents". This goal is pursued by asking 24 adult autistic participants to 24 neurotypical (NT) participants engage in a "repeated dyadic competitive game against artificial agents with calibrated reciprocal adaptation capabilities".*

*More concretely, the game includes playing against a learning machine that algorithmically adapts to the player's behavior. To win, participants' must learn to anticipate their opponent's next choice and/or try to influence it. Importantly, a cover story was used to introduce a social condition, in which participants thought they were playing against a real person.*

*1. I am very impressed by goal of the study and the methodology used to assess cognitive processes that subtend behavioral performance. However, I am less impressed by the choice of the task, because it seems to be yet another example of a task with relatively low ecological validity. I would be more excited, if someone could demonstrate how performance on such a task relates to everyday life interaction difficulties that autistic persons experience. It is nice to show that mentalizing performance is suboptimal on this task, but what if mentalizing is not the key ingredient to successfully navigating the real social environment? Please discuss this particularly in the context that your analysis predict (only) 62% of the severity of social symptoms.*

First, let us thank you for your overall positive assessment of our work. Your first comment relates to the Editor's summary (please see our response above). And we agree with you: social symptoms in AS are not solely due to mentalizing deficits. For example, they could be driven by some other issues in social cognition (including social anxiety and misperception/misunderstanding of social norms, see our response to some related point by the other reviewer below). We have now added the following paragraph in the Discussion section of the revised manuscript:

Now is our approach useful for clinical purposes? That it can achieve 79% of accuracy in diagnostic prediction is only relevant for comparing this test with other tests of the same kind, or as a proof-of-concept demonstration. In fact, the long-term goal of approaches of this kind is not to reflect the diagnosis per se (which is irrelevant), but rather to guide clinical decisions. Ideally, a useful approach should reflect a pathological mechanism and predict outcome and/or treatment response. Diagnosis is just a proxy for such prediction, and one has to admit that current psychiatric categorical diagnoses are not quite satisfactory in this regard (Insel, 2013). In turn, evaluating the clinical utility of our approach would require assessing how it relates to genetic variants, brain metrics, specific outcomes or response to intervention. This is beyond the scope of the current work, but we intend to pursue these issues in forthcoming publications. That our approach predicts 62% of inter-individual variance in social symptoms may be more interesting at first sight. This is because explaining variability beyond categorical diagnosis may be relevant for identifying clinical subcategories. But here again, establishing the clinical utility of such findings can only be done on the basis of, e.g. treatment outcome prediction. In addition, such significant but modest explanatory power is a reminder that social symptoms in AS are not solely due to mentalizing deficits. For example, they could be driven by some other issues in social cognition, including, but not limited to, social anxiety (Spain et al., 2018) or the misperception/misunderstanding of social norms (Cage et al., 2013; Izuma et al., 2011). Evaluating the relative contribution of these processes to social symptoms is clearly a promising research avenue for the computational psychiatric approach to AS.

*Other comments:*

*How do the authors explain that NT participants lose in the non-social condition, but retain earnings in the social condition? What is the role of motivation here?*

This is a critical question, and we had addressed it explicitly in our previous manuscript. But we acknowledge that this might have been buried in the SI. In brief, we think NT participants retain earning in the social framing because they can flexibly adjust their strategy, eventually relying on sophisticated mentalizing-related processes. We also think that motivation plays little role here. This is because increasing financial incentives does not change performance in the task (neither for NT nor for ASD participants). We have now modified the first paragraph of the Discussion section to highlight this point:

In line with social motivational theories of autism (Chevallier et al., 2012), one may argue that, in contrast to control participants, AS participants may not have been interested enough to invest the cognitive effort required for improving their performance in the social framing condition. Such global motivational and/or attentional interpretations are unlikely however, because AS participants actually outperform controls against 0-ToM in the social framing condition. In addition, financial incentive manipulations have no effect on performance in the game, for both AS and NT groups. This is despite the fact that both groups are consistently and equally sensitive to monetary incentives in the context of cognitive control tasks (see section 3 in Supplementary Text S1). Taken together, our results support the idea that adults with AS are not unwilling, but rather unable to exploit knowledge about the stakes of social interactions when adapting to others. However, it remains possible that social motivation might account for other aspects of autism, for example in altering the normal scaffolding of social cognition during development.

*The most interesting finding in my mind is that patients do better than NT in dealing with non-mentalizing agents. Could that be because this behavior is more 'autistic', more similar to patients' expectation? Bolis et al. have recently proposed the 'social interaction mismatch' hypothesis, which suggests that autistic persons find it easier to relate to other autistic persons (or agents). Please discuss.*

This is an interesting suggestion. We have modified the corresponding paragraph of the Discussion section as follows:

Of particular interest is the finding that autistics outperform controls in certain conditions of the game. In particular: they win against non-mentalizing learning opponents, irrespective of the task framing. Given that control participants merely achieve null earnings in the same condition, this result is a striking demonstration of the efficiency of autistics' behavioural strategy. Although strengths and peaks of ability have been reported since the first descriptions of autism as core features, they have been largely ignored in the more recent scientific literature, with few exceptions (Ostrolenk, Forgeot d'Arc, Jelenic, Samson, & Mottron, 2017). In fact, a possible explanation for such success is that non-mentalizing agents are somehow more "autistic", i.e. more similar to patients' expectations (see below). This is reminiscent to the so-called "social interaction mismatch" hypothesis, which suggests that autistic persons find it easier to relate to other autistic persons (Bolis and Schilbach, 2018; Bolis et al., 2017). In any case, future studies including measures of everyday functioning might test whether such performance peaks in the task relate to autistic strengths in real-life situations.

*With regard to the highly important topic of social reciprocity the authors do not discuss revent developments and relevant papers in social neuroscience (Schilbach et al. 2013, BBS; Schilbach 2016, Phil Trans Roy Soc; Redcay & Schilbach 2019, Nat Rev Neurosci) that have addressed this issue both conceptually and empirically.*

Well, we explicitly referred to some of the above "second-person" papers in our Introduction section, when discussing the notion of reciprocity. Nevertheless, we have now included the missing references in the revised manuscript.

*Having said all of this, I think that the paper could make an important contribution, but should be revised to address the above described comments.*

We hope that our revisions have addressed your comments adequately!

**Response to Reviewer #2:**

*In this work, the authors aim to decompose and measure reciprocal social interactions to predict ASD diagnosis. The authors present behavioral results for ASD and TD individuals performing a competitive game against artificial agents with different levels of intelligence and theory of mind. The experimental design included four different AI agents, two framing conditions (social and non-social) and two financial incentives (high vs. low). The authors main finding is that only TD individuals changed their behavior in response to the social framing, showing superior performance of TD compared to ASD when the task was framed to make individuals believe that they were competing against somebody else. Authors used the financial incentives manipulation to suggest that motivational factors did not play a role in the after mentioned framing x group observed effect. Furthermore, the authors modeled several strategies (theory of mind, RL, random choice) to explain individuals' behavior, while also modeling the possibility that participants shifted between different strategies. Modeling result suggest lower flexibility and metalizing tendencies for ASD group. The authors report 79% diagnosis classification accuracy in a leave-on-out logistic regression, and suggest that the method and modelling approach may prove useful for novel diagnostic methods for autism.*

*First, allow me to thank you for the opportunity to review this paper. I believe the use of sophisticated AI agents to objectively measure human behavioral tendencies is promising and relevant. I would like to also note that the authors expertise in modeling is well observed, and the manuscript seem to include a rich and sophisticated modeling section that, in my opinion, fits well with the journal scope and aims. However, as noted below I have a few major concerns, the main of which regards the claim that these results are valuable diagnostically. I do hope the authors will find these comments helpful, and would be happy to see further versions in the future.*

We thank you for your overall positive assessment of our work and we hope that you will find our response to your comments satisfactory.

*Major comments:*

*1. My main concern is the statement made by the authors claiming that the results can be taken as support for novel diagnosis metric. I appreciate the LOO CV result, and they are indeed impressive. However, it is my view that this reflects a large, and consistent effect within the current sample, and as such do not add much over the ANOVA results. The current study do not address within group variability or the issue of whether this method can be generalize outside the current sample (keeping in mind that ASD is a highly heterogeneous group). In that sense the CV results should be taken carefully, mostly as resonating the ANOVA results. Furthermore, the authors do not show whether symptoms severity, or specific clinical symptoms can/cannot be predicted using this approach, which might be very important. It might be also useful if the authors can find a way to shed some light on individuals that where miss classified. Did those individuals had stronger framing effect? Is there any reason to suggest they were miss-classified by the clinicians (which might actually mean this approach can add additional value to the current clinical approach)?*

Whether our approach can be deemed clinically useful is an important issue, which directly relates to the Editor's summary (and to reviewer #1's first comment). In brief, our classification results only serve as a proof-of-concept, to demonstrate that the approach provides sensible results. We agree with you that the LOO analysis is not adding much to the ANOVA, but for two exceptions. First, it shows (as you highlight), that the standard deviation of the reported effects are reasonably small (the ANOVA really cares about the standard error around the mean). Second, it helps evaluating the added value of computational phenotypes, above and beyond raw performance scores I the task (the ANOVA was performed on performance scores only). We note that we did try to make sense of misclassifications, but could not find any simple message to convey. With respect to whether we have demonstrated the ability of our approach to predict symptom severity, we respectfully disagree with you. In fact, we have shown that 62% of the inter-individual variance in social symptoms can be explained with our computational phenotypes. Importantly, these do not predict non-social symptoms, which implies that the approach's explanatory power is specific to social deficits in AS. We discuss these points in the following paragraph of the Discussion section, which we have added in our revised manuscript (it was already highlighted as a response to reviewer #1’s comments):

Now is our approach useful for clinical purposes? That it can achieve 79% of accuracy in diagnostic prediction is only relevant for comparing this test with other tests of the same kind, or as a proof-of-concept demonstration. In fact, the long-term goal of approaches of this kind is not to reflect the diagnosis per se (which is irrelevant), but rather to guide clinical decision making. Ideally, a useful approach should reflect a pathological mechanism and predict outcome and treatment response. Diagnosis is just a proxy for such prediction, and one has to admit that current psychiatric categorical diagnoses are not quite satisfactory in this regard (Insel, 2013). In turn, evaluating the clinical utility of our approach would require assessing how it relates to genetic variants, brain metrics, specific outcomes or response to intervention. This is beyond the scope of the current work, but we intend to pursue these issues in forthcoming publications. That our approach predicts 62% of inter-individual variance in social symptoms may be more interesting at first sight. This is because explaining variability beyond categorical diagnosis may be relevant for identifying clinical subcategories. But here again, establishing the clinical utility of such findings can only be done on the basis of, e.g. treatment outcome prediction. In addition, such significant but modest explanatory power is a reminder that social symptoms in AS are not solely due to mentalizing deficits. For example, they could be driven by some other issues in social cognition, including, but not limited to, social anxiety (Spain et al., 2018) or the misperception/misunderstanding of social norms (Cage et al., 2013; Izuma et al., 2011). Evaluating the relative contribution of these processes to social symptoms is clearly a promising research avenue for the computational psychiatric approach to AS.

*Second, the current test provides, like almost all measures in the field, an assessment of some symptoms that should be assumed to be predicted by ASD (not vice versa). I agree that a task-based objective measure could facilitate symptom-based diagnosis, which is currently done only by interviews and self-report. However, I think the authors need to specify how exactly this could be helpful. My understanding is that in the current sample the classification accuracy is not much worse than clinical measures. Given that task based estimates tend to be in general much less reliable compared to self-report measures (e.g., Enkavi et al., 2019), we can only assume that in the long run, it might be very difficult to justify the use of such approach as a clinical measure.*

This is a fair comment, which applies to most cognitive/computational studies of this kind. In particular, we acknowledge that we cannot evaluate the test-retest reliability of our task-based measures (including computational phenotypes) on the current population sample. However, we are currently conducting a large sample study based on an online/smartphone version of the task to address this and related issues (see <https://sites.google.com/site/brainus1en/>). Although the recruitment of AS participants progresses at a slow pace (current enrollment includes about 32,000 control subjects and about 40 ASD patients), this will allow us to perform confirmatory analyses and quantify the psychometric properties of our approach. In particular, we will be in a position to evaluate the interest of the approach when used as a screening tool. Would this first proof-of-concept paper be eventually published, we would report on these concerns in forthcoming publications.

*2. ASD individuals might have unpleasant memories from social interactions, more compared to TD individuals. Therefore, is it possible that for the ASD group the social version is less engaging, leading to worse performance compared to TD? I appreciate the fact that the authors manipulated reward magnitude, yet this might be somewhat unrelated since the claim here is that ASD might have been more anxious in the social condition, lowering their performance (What could really help in future studies is a social relevant clinical control group like individuals with social anxiety).*

It is interesting to raise the question of social anxiety, a condition frequently associated to AS. Here, autistic people behave similarly in social and non social framing conditions, suggesting that their perception of the social nature of the task (as reflected in debriefing) doesn't translate into distinct strategies. It is not impossible that this apparent indifference stems from anxiety that would preclude the use of specifically sophisticated, social, strategies. However, it is barely the most parcimonious interpretation, especially if one considers that motivation does not affect strategies and that no participant reported anxiety during the task. Nevertheless, we agree that the effect of social anxiety on the cognitive strategies used in social interaction is an important question that will be addressed in a future (ongoing) study.

*3. The authors use a correlational analysis to support the fact that the ASD group show no framing effect, which is essentially a null result. While the group x framing interaction might be enough here, I am not sure I understand why the correlational analysis in the SI, section 4 helps with NHT issues regarding the simple effect of framing for the ASD group. Since the whole distribution can be shifted between conditions, the correlations can be high or low regardless of whether a simple framing effect is present within the ASD group. I would suggest that the authors use a Bayesian ANOVA, and report effect-size measures such as partial et-square (or any other) to demonstrate the main result – no framing effect for ASD compared to TD.*

This is a good point. For the sake of simplicity, and since this is not critical for our main message, we have decided to simply remove this part from the Supplementary Text S1 (and we have removed any mention of this analysis in the main text).

*4. Is it possible that the k-ToM model fits the ASD better, thus leading to lower flexibility? If this is the case, is it possible that ASD find it harder to play the task due to the fact that the AI agent is more suitable opponent for their strategy? Finally, could this mean that they do not show framing effect for high k opponents, due to the fact that the game is (much) more difficult for them, not leaving much room for improvement?*

The computational model that fits the strategy of AS participants best is the “influence learning” model. More precisely, this model seems to provide quite a stable explanation for their behaviour, across framings and repetitions, which underlies their lower flexibility score as well as the absence of a framing effect. It also explains why their performance is low against mentalizing AI agents (1-ToM and 2-ToM). However, we would not say that AS participants find the game more difficult. For example, they perform significantly better than controls against non-mentalizing agents (in both social and non-social framings). Note that this is also explained by the fact that they may rely on “influence learning”. We have modified the corresponding paragraph of the Discussion section accordingly:

In contrast, trial-by-trial choice sequences of most AS players, in both framing conditions, are captured by a single model, namely: "influence learning" (Hampton et al., 2008). From a computational standpoint, this model possesses broad adaptive fitness because it essentially is a generic way of dealing with environments that react to one's actions (Devaine et al., 2017). In other words, influence learning can be seen as an all-purpose cognitive toolkit that would be expected to perform well in a wide range of contexts, excluding competitive interactions with mentalizing agents (cf. pattern of performances against RB, 0-ToM, 1-ToM and 2-ToM in section 8 in the Supplementary Text S1). Note that this explains why AS participants perform better than NT controls against non-mentalizing agents (in both framing conditions), and why they show worse performance against mentalizing agents (in the social framing condition). That they rely on influence learning in both framings also explains their lower flexibility score, as well as the absence of a framing effect on raw performance. Strictly speaking, an agent capable of influence learning is thus not "mind blind", but it cannot adjust its behavioural strategy to the intentions of mentalizing agents. In other words, even if equipped with a sophisticated perceptual apparatus (that would enable the recognition of ecological social signals), an influence learner would show limited social reciprocity. Reliance on this -or similar- adaptation strategy thus provides a computational explanation for an important aspect of the autistic social phenotype, which would not depend upon motivational factors and/or cognizance of the social context.

*5. Given the 1.3 response deadline, is it possible that the ASD (which can show prolonged RTs) had to lower their response threshold, and suffered from less optimal speed-accuracy trade-off point?*

We think this is unlikely, for two reasons. First, AS and NT participants show no significant difference in IQ, including measures of processing speed. Second, AS participants perform significantly better than controls against non-mentalizing agents, in both social and non-social framing conditions…

*6. I think the results regarding flexibility are not explained with enough data. Is there any way the authors can outline the fit of the different models, and give the reader some understanding of which models where used by which groups? What is the relationship between flexibility parameter and overall model fit?*

Well, we had already reported the estimated frequency of computational models, separately for each group of participants and each framing condition. Please refer to section 5 (or section 6 in the previous version of the manuscript) of the Supplementary Text S1. In brief, AS participants rely on “influence learning”, whereas no model clearly wins for NT controls. Note that the latter result does not imply that models do not fit trial-by-trial NT participants’ data. Recall that we had included a chance model in the comparison set, namely: “Biased Nash”. This model reduces trial-by-trial choice data to a sequence of (possibly biased) binomial random samples. This is as close a null model as it gets. However, it very rarely wins the within-subject model comparison, since its average frequency is below 20%. In other terms, other models are better than chance in about 80% of the cases. This implies that the flexibility score is not reducible to models’ fit accuracy. We have added the following paragraph in the Supplementary Text S1 (section 5):

One can see that no learning model clearly stands out for the NT group. In fact, Bayesian omnibus risks show that the model frequency profiles of Figure A3 do not deviate strongly from expectations derived under chance (social framing: BOR=0.96, non-social framing: BOR=0.44). This does not imply that models do not fit trial-by-trial NT participants’ data. Recall that we had included a chance model in the comparison set, namely: “Biased Nash” or BN. This model reduces trial-by-trial choice data to a sequence of (possibly biased) binomial random samples. This is as close a null model as it gets. However, it very rarely wins the within-subject model comparison, since its average frequency is below 20%. In other terms, other models are better than chance in about 80% of the cases. Rather, high BORs imply that RFX-BMC cannot conclude on any winning learning model. Note that this null finding is not inconsistent with the fact that NT participants' learning repertoire exhibit high flexibility across conditions.

*7. Minor comments:*

*- The author describe former ToM tests as having 'unreliable results and poor psychometric properties' (bottom, pg3). I think it would be great if the authors could specific more exact reliability/validity estimates, and show where possible how the current test differ, if they are to keep the claim that this method is proved to be clinically valuable.*

As you have seen from our modified Discussion section, we have toned our clinical claims down. And as you may recall, the complete sentence that you are referring to highlights practical issues mostly with adult individuals:

ToM impairments have been repeatedly evidenced in autistic children using tests of, e.g., false belief understanding (Frith and Happé, 1994; Girli and Tekin, 2010; Grant et al., 2001), sarcasm/irony detection (Wang et al., 2006; Zalla et al., 2014) or moral evaluation (Fadda et al., 2016; Margoni and Surian, 2016). However, these tests yield quite unreliable results and have poor psychometric properties in older individuals (Pinkham et al., 2018), including ceiling effects in adolescents and adults (Happé, 1995; Ponnet et al., 2008). This is why, although theoretically relevant to autism, quantitative tests of ToM has had only limited impact on diagnosis or intervention to date (Fletcher-Watson et al., 2014).

We hope that you will agree with us that this statement is neither unfair, nor irrelevant for motivating our study. Although we could provide a quantitative range for most psychometric properties (e.g., test-retest reliability) of established ToM tests, we could not compare these with our approach yet. We note that we are currently evaluating the psychometric properties of our approach (cf. response to your comment above). When proceeding with the publication of those results, we will report the psychometric improvement of our approach, if any. We simply do not have the data to answer these questions yet.

*- I understand the need to keep some results out of the main text, but please clearly note the full design in the main text (including the reward manipulation) so the reader will know what to except.*

You are referring to the fact that we did not mention the incentive manipulation in the main text. This simplification was a deliberate choice of ours, motivated by the absence of a motivational effect in our study. Having said this, we acknowledge that this might have been a mistake and we have now modified the Methods section accordingly:

The behavioural task consists of a computerized game (60 trials each) with two framing conditions. In the *social* condition, the task was framed as an online competitive game with someone else. In the *non-social* condition, it was framed as a gambling game. In fact, both games were played against four different learning algorithms with different artificial mentalizing sophistication (ranging from a random sequence with a bias to so-called *2-ToM* agents: see below). Note that, on top of framing and opponent factors, we also varied the financial payoff attached to a correct answer in the games. More precisely, the maximal payoff that participants could earn over one game session was either 10$ (high reward condition) or 1 cent (low reward condition). This manipulation, however, did not induce any effect (cf. section 3 of the Supplementary Text S1). In what follows, we thus refer to this experimental factor as a repetition of the task conditions. At each trial, subjects had 1300 ms to make a binary choice (the place to hide or the slot machine to try), which was fed to the learning algorithms to compute online predictions of the participant's action at the next trial. In total, each participant performed 2×4×2=16 games (2 framings, 4 opponent types, 2 repetitions) in a pseudo-randomized order. We refer the interested reader to the Supplementary Text S1 for more details regarding the experimental protocol.

*- Fig 1 – I'm not sure I understood why use a net rate rather than the more straight forward accuracy rate?*

The net rate is such that it is null when participants are at chance level (50% choice accuracy). This simplifies the visual interpretation of performance patterns. Note that we have now modified Figure 2’s caption accordingly:

**Figure 2: Behavioural performance results.** Group average net rate of correct answers (y-axis) against the four opponent types (x-axis) for both framing conditions (blue: social, red: non-social) in both AS (left) and control (right) participants. Note: The net rate of correct answers is defined as (n_c_-n_i_)/(n_c_+n_i_), where n_c_ and n_i_ are the number of correct and incorrect responses, respectively. Hence, it is null when participants perform at chance level (50% accuracy). In this and all subsequent figures, error bars depict the standard error around the mean.

*- The authors report in the first paragraph of the discussion four analyses to support their claims yet three of which are tacked in the SI, which seems to be written with less care. Is there any way to improve the presentation?*

First, note that we have removed any mention to the analysis of correlations between performance patterns across framings. And two of the remaining analyses are in fact described in the main text. This leaves one out of three analyses in the Supplementary Text S1 (namely: Volterra decompositions of trial-by-trial choice sequences). In addition, we have modified the corresponding section of the Supplementary Text with the aim of improving its readability. We hope you will find it both clear and concise enough.

*- The authors suggest that a completely random strategy is "embarrassingly simple". I would argue that asking a human individual to act in a perfectly random manner is rather difficult, and against some human opponents could be useful to shift to. In that sense, it is interesting to see what role this strategy played in the individuals data.*

As we highlighted when responding to your previous comment regarding the possible relationship between the flexibility score and mode fit, we included the random strategy in the model comparison set. Section 5 of the Supplementary Text S1 shows that it explains about 20% of NT participants’ data (cf. Figure A3), and less than 5% of AS participants’ data (cf. Figure A4).

*Typos:*

*- SI section 4:" whether there are there",*

This section has been removed (cf. responses above).

*- "Figure A2 below show the correlation between" I think you meant Fig A3*

Same response.

*- " (Fig A3, right inset) analyses show a significant correlation between framings for the AS group" I think you meant between repetitions.*

Same response.

*- Please carefully proof the SI as it seems to include valuable parts of the study.*

Thank you: we have done this and we hope you will think that the literary quality of the SI has improved enough.

1. <https://www.nimh.nih.gov/about/directors/thomas-insel/blog/2013/transforming-diagnosis.shtml>
