# Supplementary material for: Social behavioural adaptation in Autism
Source: PLoS Comput Biol. 2020 Mar 16;16(3):e1007700. doi: 10.1371/journal.pcbi.1007700 (PMC7108744; doi:10.1371/journal.pcbi.1007700)
Supplement: Attachment — Submitted filename: response to reviewers.docx [file pcbi.1007700.s003.docx]

**Editor's comments:**

*I would like to comment on the points still raised by Reviewer 2, and ask that you kindly respond in the Discussion to their first point, which regards response times. I think that Reviewer 2's second point, although of some validity, will not be a serious concern for our readership. The term 'clinical utility' may be changed, for example, to 'clinical relevance' at the author's discretion, but otherwise the Discussion (and slightly less so the Introduction) now make it clear that the paper makes an incremental contribution towards 'bedside' tests for AS, rather than any exaggerated claims. However, I would kindly ask the authors to address the point regarding response times. I feel that Reviewer 2's hypothesis is rather too elaborate, requiring that AS participants were slower in general, but not so slow as to make more mistakes in the nonsocial condition where they performed better, yet too slow for the social condition. Reviewer 2 does have a point, that high-ToM may be too costly, in terms of response time, for AS participants rather than then being altogether unable to consider it. Therefore, when they attempt such a high-ToM strategy their performance deteriorates and may resemble 'Influence' decision-making. However I feel that RT analysis is outside the scope of this paper and should not delay its publication. I would therefore suggest that the authors address Reviewer 2's suggestion that RT analysis would make conclusions more compelling by discussing it in an additional paragraph on future research directions. Response time analysis - among other methods - may indeed move understanding from the algorithmic level of this paper to one incorporating neural implementational features (such as accumulator processes that generate response times) and this may be addressed in future work.*

We thank you for your kind and patient support. We have modified the manuscript to tone down (even further) the claims regarding the clinical utility of our work. In particular, we have followed your advice and now systematically speak about clinical *relevance*. We have also conducted a quantitative analysis of RT data (which we added in the revised Supplementary Text), and have included a related paragraph in the Discussion section of our revised manuscript (cf. response to R2's first comment below). Note that we also inserted an additional paragraph in the Discussion section, where we comment on the validity of the task for assessing social constructs such as "contact" and "reciprocity" (with which we introduce our work). It is copied below:

One may also question the nature of the social cognitive processes that the task assesses. Although our original intention was to address the elusive notions of contact and reciprocity, the task itself falls short of a few important features of real-life social interactions. For example, it ignores the diversity of social signals (e.g., verbal/body language, facial expressions) and modulatory factors (e.g., in-group/out-group context, familiarity) that are relevant for establishing contact with others. It also does not involve changes in others' intentions (e.g., competitive/cooperative) and/or attitudes (e.g., friendly/aggressive, dominant/submissive), which would be necessary to assess certain aspects of social reciprocity. Instead, it focuses on peoples' ability to respond and/or influence others' actions in a simplified competitive setting. Clearly, this cannot account for the breadth of social cognitive processes that underlie contact and reciprocity. One might even think that task performance may not load very heavily on social cognitive processes, when compared with other instrumental processes (e.g., working memory or reasoning). This is unlikely, however, given that we have shown, in a very large online population sample, that a very small amount of inter-individual variance in the game's performance can be predicted from cognitive control skills (Klindt et al., 2017). In any case, we think the simplicity of our task design also has its virtues. This is because it eventually enables us to construct a non-social control condition that is matched with the social condition in terms of goal-relevant information (cf. trial-by-trial feedbacks). This turns out to be critical to discriminate between AS and NT participants.

**Response to Reviewer #1:**

*The authors have satisfactorily addressed my previous comments in their revision..*

Thank you.

**Response to Reviewer #2:**

*Dear Authors, Thank you for your answers - much appreciated. Please find below two followup issues. Many thanks. R2.*

Thank you.

*1. Given the 1.3 response deadline, is it possible that the ASD (which can show prolonged RTs) had to lower their response threshold, and suffered from less optimal speed-accuracy trade-off point?*

*"We think this is unlikely, for two reasons. First, AS and NT participants show no significant difference in IQ, including measures of processing speed. Second, AS participants perform significantly better than controls against non-mentalizing agents, in both social and non-social framing conditions…"*

*Thank you for your reply, but I am not convinced. A response deadline of 1300ms is very strict and could majorly affect decision threshold. ASD are known to show prolonged RTs and increased RTV (mostly a higher rate of very slow RTs, and mostly due to co-occurrence with ADHD symptoms). I would encourage the authors to give this more thought. For example, it might be that with higher mentalizing agents (RB to 2-TOM) all individuals required more time to make a decision. Given that ASD are assumed to be slower in general, the strict RT deadline might render the high TOM conditions much more difficult for them, which can then reduce their ability to show sensitivity to the framing effect. An RT analysis, given this strict response deadline would thus make your model-free analysis much more compelling. PS pencil-paper test is not enough to indicate no RT differences between the groups as it does not have the required sensitivity.*

This is a good point. We acknowledge that differences in cognitive speed may induce performance differences in situations where "good" decisions may take more time. Indeed, AS participants are, on average, slightly slower than controls (see Figure 1 below). They, however, do not show a higher rate of behindhand responses (i.e. trials whose RT reaches the decision time limit).

Figure 1. Empirical histogram of reaction time for both AS (red) and control (blue) groups. Note that the truncated RT peak on the right tail of the RT distributions corresponds to behindhand responses.

Nevertheless, we conducted a quantitative analysis of behindhand response rates, which we have included in the Supplementary Text of our revised manuscript. In brief, the pattern of behindhand responses is globally inconsistent with the performance pattern reported in the main text. We have also added a new paragraph in the Discussion section, which we have copied below:

One may ask whether the performance pattern we report here may not be due to the fact that AS individuals are typically slower than neurotypic people. This would be because in the task, participants have only 1.3 second to respond, which would potentially be too short for AS individuals to reach the correct decision. The ensuing "behindhand errors" could then confound analyses of performance data. In particular, this would explain performance differences in situations where strategic thinking is (in principle) most needed, i.e. against mentalizing agents in the social framing condition. We find that this is an unlikely confound however, because the pattern of behindhand responses (i.e. responses whose RT reaches the timing threshold) and performance are globally inconsistent with each other (cf. section 4 of the Supplementary text S1). Nevertheless, we acknowledge that dual analyses of concurrent performance/RT data (such as those based upon accumulation-to-bound models that generate both choice and RT data) may indeed provide insights into the neural implementational processes underlying our behavioural results. This may be addressed in future work.

*2. In my previous comments, I suggested that my main concern is the statement made by the authors claiming that the results can be taken as support for novel diagnosis metric. I appreciate the authors changes in the study, but it is my personal view that this paper still creates strong expectations for the reader regarding clinical relevance. For example, the authors note in the first paragraph of the intro that “This work evaluates the clinical utility of a computational decomposition...”, and mention the need for a ‘true test’ for ASD..*

We understand your comment and have now changed the wording of this and related paragraphs. For example, we have replaced the notion of "clinical utility" to that of "clinical relevance". We hope you will find these modifications satisfactory.
